# Beyond concreteness: why word specificity is the missing piece in theories on embodied language comprehension

**DOI:** 10.3389/fpsyg.2025.1718655

**Published:** 2025-11-25

**Authors:** Marianna Bolognesi

**Affiliations:** Department of Modern Languages, Literatures and Cultures, University of Bologna, Bologna, Italy

**Keywords:** abstraction, specificity, concreteness, abstractness, semantic categorization, embodiment

## Abstract

The concreteness effect has long been associated with embodied theories of language, which propose that concrete words are easier to process than abstract ones because they more directly engage perceptual and motor simulations. However, empirical findings on this effect remain mixed. This paper argues that such variability stems from overlooking a crucial semantic dimension: word specificity. Drawing on evidence from the ERC-funded *ABSTRACTION* project, I defend (based on classic and more recent empirical studies) that specificity, defined as a word's position within a conceptual hierarchy and corresponding to the inclusiveness of its category, plays a key role in shaping lexical access and conceptual organization, alone and in interaction with concreteness. The relationship between these two dimensions, and its implications for embodied language processing, has so far remained largely unexplored. Integrating specificity into models of embodied semantic representation offers a more nuanced account of how language supports both abstraction and embodiment in cognition.

## 1 Concreteness and the embodiment of language

For years, the contrast between concrete and abstract concepts has occupied a central place in psycholinguistics and cognitive science (see Bolognesi M. Steen G., 2018, 2019 for reviews). Perhaps the most widely documented phenomenon in this domain is the *concreteness effect*, the advantage that concrete words show over abstract ones in tasks of processing and memory (Paivio, 1991; Schwanenflugel, 1991; see Huang and Federmeier, 2015 for a review). For example, people typically recognize or recall words such as *dog* or *table* faster and more accurately than words like *freedom* or *idea*. The dominant explanation is that concrete concepts benefit from richer sensorimotor grounding: they can be anchored in perceptual and motor experience, making them easier to access and manipulate during comprehension and memory retrieval (e.g., Jessen et al., 2000).

The claim that understanding language involves the activation of perceptual and motor systems is a cornerstone of embodied and grounded cognition accounts (e.g., Barsalou L, 1999; Pecher, 2005; Glenberg A, 2010). According to this perspective, understanding the word *kick* involves partial re-enactment of motor routines, while processing the meaning of *red* recruits visual representations of color. A first milestone in the debate came with an international conference organized by de (de Vega and Glenberg 2008). The edited volume that collected the debate brought together contrasting perspectives: proponents of embodied cognition argued that meaning is grounded in perceptual and motor simulations, whereas advocates of symbolic approaches insisted on the need for abstract, amodal representations to account for language's flexibility and generativity. Many symbolic theorists drew on the distributional hypothesis (Harris, 1954; Firth, 1957), which holds that a word's meaning can be inferred from its linguistic contexts. Interestingly, this view laid the groundwork for more efficient distributional semantic models and, ultimately, today's large-scale language models tha drive generative AI. The exchange created a productive tension that has since shaped research efforts to reconcile these positions, emphasizing both the embodied grounding of meaning and the symbolic mechanisms that enable us to move beyond direct experience.

Within this debate, a persistent challenge for embodied theories of language remained precisely the so-called *bottleneck of abstract concepts*: whereas concrete concepts are linked to tangible referents that can be directly processed through sensory modalities, abstract notions such as *freedom* or *belief* lack perceptual anchors, making it less clear how they could be simulated in sensorimotor systems to the same extent. To address this challenge, various embodied accounts have been proposed, all maintaining that abstract concepts remain grounded, albeit through different mechanisms (see Pecher, 2018, for an overview). One line of work argues that abstract concepts draw more heavily on alternative embodied resources, such as affective experience and social interaction (Borghi et al., 2017; Kousta et al., 2011). For instance, concepts like *justice* or *democracy* may be less tied to perceptual imagery and more to emotional and communicative contexts in which they are encountered. Another proposal emphasizes metaphorical mappings as the indirect grounding of abstract concepts (Lakoff and Johnson, 1999). For instance, a concept like *justice* may be metaphorically understood in terms of balance, represented by a scale. A further account, originally advanced by Barsalou and and Wiemer-Hastings (2005) and later supported empirically by McRae et al. (2018), suggests that both concrete and abstract concepts rely on situated simulations, though with different emphases: concrete concepts are mainly defined by entity-related features and perceptual properties (taste, color, and so forth), whereas abstract concepts, lacking a primary referent, are organized around situational elements such as participants, actions, and settings.

Following the attempts in the 2000s and early 2010s to tackle the abstractness bottleneck through affective grounding, metaphorical mappings, and situated simulations, the debate entered a new stage. A second milestone was the #symbodiment initiative and the related special issue introduced by Mahon and Hickok (2016), which revisited the symbolic vs. embodied divide with a more integrative outlook. This project organized the discussion around different central questions: the format of conceptual representations, the content they encode, their neural and cognitive organization, and the dynamics of how conceptual and sensorimotor systems interact. Instead of rehearsing old oppositions, the focus shifted to making explicit the underlying assumptions that connect sensorimotor mechanisms with higher-level conceptual knowledge. The project also called for methodological pluralism, bringing together behavioral experiments, neuroimaging, computational modeling, and corpus-based analyses to provide converging evidence on how symbolic and embodied processes can be reconciled within a unified account.

Overall, these endeavors sparked growing interest in moving past the simplistic concrete-abstract divide when considering the embodied basis of language processing and representation. In fact, despite extensive study, the binary opposition between abstract and concrete concepts proves restrictive and sometimes yields conflicting findings. For example, although concrete words are generally thought to be learned earlier than abstract ones, children can acquire certain abstract terms at an early stage (Ponari et al., 2018). Likewise, while concrete words are typically processed more quickly than abstract words, adults show faster processing for some abstract concepts than for others (Kousta et al., 2011). Such evidence indicates, first of all, that abstract concepts are not a homogeneous category but can be analyzed and divided into various subtypes (Villani et al., 2019), where each subtype may rely on distinct affective, linguistic, or situational information and therefore afford a different type of grounding. Secondly, these points suggest that we might be overlooking some important aspects of what is packed into the notion of concreteness.

Therefore, before setting the concrete-abstract dichotomy aside, it might be helpful to remember why it has proven so persistent and what can be done to refine it. The abstract-concrete distinction is not simply an artifact of experimental methodology, but reflects long-standing intuitions in philosophy, psychology, and linguistics, and it is so ingrained that it is even taught in primary and middle school curricula. Recognizing these historical roots is essential, and so it is understanding better what we mean when we say “concrete” and what we mean when we say “abstract”.

## 2 Operationalizing concreteness

The most common way of operationalizing the intuitive notion of concreteness is through concreteness ratings, which have been collected through crowdsourcing tasks across several languages, including English (Brysbaert et al., 2014a), Dutch (Brysbaert et al., 2014b), Italian (Montefinese et al., 2014), Spanish (Guasch et al., 2016), Croatian (Peti-Stantić et al., 2021), and others. In these studies, participants are asked to judge isolated words on Likert-type scales, typically ranging from 1 (very abstract) to 5 or 7 (very concrete), thereby providing a numerical estimate of how concrete or abstract a word is perceived to be. These resources have become indispensable for psycholinguistic research, supporting large-scale experimental and computational investigations of semantic processing across languages.

Despite their widespread use, concreteness ratings have important methodological limitations. For instance, Pollock (2018) has demonstrated that the mean values reported in large-scale norming studies often obscure substantial variability in participants' judgments. In particular, at the extremes of the scale, ratings are usually stable and consensual: words such as *table* or *banana* are consistently judged concrete, while *freedom* or *justice* are reliably rated abstract. Difficulties arise with words that fall in the middle of the scale, where judgments diverge sharply. For instance, *game* or *side* can elicit both concrete interpretations—*game* as a physical activity, *side* as a tangible surface—and more abstract ones, such as *game* as a strategic or social activity and *side* as a metaphorical aspect of a problem. This may result in mean concreteness ratings that rest in the middle of the Likert scale, hiding a high standard deviation.

These challenges are motivated by semantic phenomena such as ambiguity, polysemy and in particular by metaphorical extension (a very productive mechanism to generate new word meanings). Many words with a basic concrete sense can develop abstract interpretations through metaphor. For example, *field* may refer to a physical expanse (*a field of flowers*) or to an abstract domain (*a field of research*). Typically, the extended metaphorical meaning is much more abstract than the original literal meaning. Reijnierse et al. (2019) showed that concreteness ratings are strongly influenced by which sense of a word participants activate. When contextual information is provided in a sentence that disambiguates literal from metaphorical usage, judgments become much more consistent: literal readings are reliably rated as concrete, while metaphorical readings are consistently rated as abstract. Conversely, when presented in isolation, words may lead raters to conflate multiple interpretations, some concrete, others abstract, so that the resulting average does not faithfully represent any single meaning. A recent endeavor (the ConcreText project) addressed this problem by providing a (limited) dataset of concreteness ratings for English and Italian words embedded in sentences, thereby capturing context-sensitive variations in perceived concreteness (Montefinese et al., 2023).

Overall, these findings indicate that although concreteness ratings are an invaluable tool for large-scale semantic research, they must be interpreted with caution. What appears as a single score may reflect the conflation of judgments on multiple senses of the same word.

Beyond the distinction of whether a concept has a perceptual referent, concepts also vary in the sensory modalities (vision, audition, touch, smell, taste, interoception) and motor systems with which they are most strongly associated. Concreteness, however, collapses this richness into a single scale, thereby oversimplifying conceptual representation. A more accurate account of embodiment requires norms that capture the distribution and intensity of sensorimotor experiences across modalities. To this end, Lynott et al. (2020) introduced large-scale ratings of perceptual and action strength for thousands of words across multiple modalities (vision, hearing, touch, taste, smell, interoception, and motor dimensions). Previous attempts in this direction were also provided, on smaller datasets (e.g., Della Rosa et al., 2010; Lynott and Connell, 2009). Extending this idea that concreteness ratings should be unpacked into more fine-grained dimensions, Troche et al. (2017) conducted a study aimed at setting a “topography” of word concreteness, in which participants evaluated words and concepts across a wider range of cognitive dimensions, including color, motion, emotion, valence, and space. Their analysis revealed three overarching clusters of factors (endogenous, exogenous, and magnitude) supporting the view that abstract and concrete concepts are not represented by two separate systems, but instead fall along a continuum within a unified, multidimensional semantic space.

These endeavors lead to the following open question: are there more variables (packed in or conflated with concreteness) that contribute to the perceived difference between concrete and abstract concepts and in general to the concreteness effect, and that must be considered when discussing the overall embodied nature of semantic representation?

## 3 The elephant in the room: categorical specificity

A crucial dimension in debates on conceptual representation is *categorical specificity*, or the degree of detail with which a word defines a conceptual category, positioning it in a relevant conceptual hierarchy. Specificity distinguishes between superordinate concepts, which cover broad domains such as *animal* or *emotion*; basic-level concepts, which provide more prototypical anchors like *dog* or *happiness*; and subordinate concepts, which denote highly detailed instances such as *beagle* or *schadenfreude*.

From a cognitive perspective, classic empirical evidence supports the notion that basic-level concepts occupy a privileged status in human categorization and semantic processing. Behavioral studies have long shown that basic-level terms are recognized, produced, and learned more efficiently than either superordinate or subordinate ones (e.g., Rosch et al., 1976; Rosch, 1978). Moreover, recent neurocognitive work has confirmed and refined this picture: electrophysiological and neuroimaging data indicate that basic-level distinctions emerge earliest in the time course of object recognition, and that their neural representations dominate even when the task requires access to subordinate categories (Greene and Rohan, 2025). This suggests that the basic level provides an automatic, default grain of conceptual organization, likely reflecting its optimal balance between informativeness and cognitive economy (Bauer and Just, 2017). By contrast, superordinate concepts appear to demand greater executive and semantic control and appear to be learned later by children (Rosch et al., 1976; Singer-Freeman and Bauer, 1997; Schumacher et al., 2009). Because they group together items with fewer shared perceptual features, their processing is often slower and less robust (something we reported also in Lamarra et al., 2025, a study described in the next section).

At the other end of the hierarchy, subordinate concepts are more fine-grained and perceptually detailed, but also less generalizable. They are often accessed more slowly than basic-level categories, unless perceptual distinctiveness or domain expertise shifts the balance in their favor. For experts, for instance, subordinate distinctions often become as accessible and natural as basic-level ones: dog breeders identify a *beagle* as readily as a layperson identifies a *dog* (Tanaka and Taylor, 1991). Similarly, when subordinate categories are highly perceptually distinctive, such as *penguin* or *giraffe*, they may be processed with the same ease that is usually attributed to the basic level (Jolicoeur et al., 1984). The boundary between basic and subordinate levels is therefore not fixed but rather depends on the perceiver's knowledge, experience, and salience cues in the environment. In general, I argue, the distinction and assessment of levels of conceptual representation may be better operationalized on a fine-grained scale, rather than on a coarse tripartite differentiation between superordinate, basic and subordinate.

Furthermore, while semantic categorization levels have been extensively investigated in relation to concrete categories, their application to abstract concepts has been virtually absent, partly because abstract concepts were traditionally assumed to lack a hierarchical organization. Yet, intuitively, abstractness also unfolds across levels of specificity, ranging from broad notions such as *justice* or *belief* to more specialized forms like *restorative justice* or *religious belief* . Language plays a key role in this process: in the example above, it is through linguistic compositionality that increasingly fine distinctions are carved out within broad domains. A general concept like *freedom* can be narrowed into *freedom of speech* or *academic freedom*, each with its own social and normative scope. Likewise, *justice* can be specified as *restorative justice* or *distributive justice*, while *belief* can be linguistically refined into *religious belief* or *political belief* , and so forth. Linguistic compositionality, however, is not a peculiarity of abstract concepts alone: it is pervasive also within concrete domains, and it operates in the creation of compounds such as *apple cake*, as a subtype of *cake, blueberry* as a type of *berry*, and so forth. Additionally, composition is not the only mechanism abstract concepts deploy to expand within the hierarchical structure of semantic categories: the abstract concept *religion* illustrates this particularly clearly. *Religion*, a subtype of *belief*, is a quite general category, as it can be specified into major traditions such as *Monotheism* and *Polytheism*, and into more fine-grained categories such as *Christianity, Islam*, or *Buddhism*. Each of these types can be further differentiated into denominations or sects, including *Catholicism, Protestantism* (types of Christianity), or *Zen* (type of Buddhism) and into even more fine-grained ones, like *Calvinism* and *Methodism* (types of *Protestantism*). This example illustrates that, just as many concrete concepts are structured hierarchically, (at least some) abstract concepts too are organized into very rich and multi-layered taxonomies (Villani et al., 2024), in which very specific categories are lexicalized into specific words rather than being obtained through linguistic composition. The granularity of some abstract concepts may be peculiar of concepts that belong to social reality, and of categories that exist as collective constructs, sustained by cultural practices and discourse, but this remains so far an open empirical question. For the scope of the present article, I suggest that the hierarchical organization of these types of abstract concepts may reflect how language both creates and stabilizes social entities, carving out distinctions that persist because communities acknowledge and reproduce them. In this sense, language-mediated abstraction (operationalized as specificity) is both a cognitive resource and a social mechanism that structures layered conceptual and social hierarchies and anchors abstract domains in shared cultural life.

In cognitive linguistics, the notion of specificity has been extensively theorized under its complementary concept of *schematicity* (Langacker, 1987, 2008; Clausner and Croft, 1997; Tuggy, 2010). Within the framework of Cognitive Grammar, schematicity refers to the degree of abstraction or precision with which a conceptual structure is represented. A *schema* is a superordinate construct that captures the common features shared by a set of more specific elaborations or instantiations. As (Langacker 1987: 132–135) explains, schematicity “pertains to level of specificity, i.e. the fineness of detail with which something is characterized,” such that a schema provides less information and is compatible with a wider range of options. Meaning construction in this view involves continuous movement along the specificity hierarchy, and linguistic expressions can be characterized by their relative level of schematicity. As a matter of fact, Langacker's approach highlights that specificity is not merely a descriptive label for lexical granularity, but a cognitive dimension governing the construal of conceptual content and the relationships among linguistic forms.

Similarly, Croft and Cruse (2004) emphasize that specificity is a gradient property intrinsic to conceptual structure. Within their model, conceptual and linguistic schemas form taxonomic networks, where each node represents a level of abstraction, and instantiation relations define category membership. Their notion of *default specificity* (Croft and Cruse, 2004) captures the tendency for linguistic expressions to be interpreted at a prototypical level of granularity unless contextual factors impose greater generality or specificity. This dynamic view underscores that specificity is context-sensitive and closely tied to processes of categorical construal and conceptual alignment.

Further insight into specificity was provided by Murphy and Lassaline (1997), who examined how category hierarchies guide conceptual organization and generalization. Their classic work demonstrated that people reason and learn differently at distinct levels of abstraction, and that the accessibility of category information depends on its position within a conceptual taxonomy. These findings align with the cognitive linguistic notion that the mental lexicon mirrors conceptual hierarchies, and that lexical access involves movement along these hierarchies depending on discourse demands.

Finally and most importantly, the distinction between specificity and concreteness has also been discussed in the context of Conceptual Metaphor Theory (Lakoff and Johnson, 1980). As Dancygier and Sweetser (2014: 43) compellingly observe, “specificity and concreteness are two quite different parameters. *Tree* and *black oak* are equally concrete object names, but *black oak* names a more specific and elaborated subcategory of the more general category of *trees*. Similarly, *ponder* is more specific than *think*, though both are equally abstract.” Within metaphorical constructions, specificity governs how mappings are organized hierarchically: broad metaphors (e.g., ARGUMENT IS WAR) can be instantiated by more specific mappings (DEFENDING A POINT IS BLOCKING AN ATTACK). Conversely, concreteness differentiates source and target domains, with source domains tending to be more concrete and target domains more abstract (Dancygier and Sweetser, 2014). Furthermore, within the framework of Conceptual Metaphor Theory, the notion of *schematicity* has been captured through the construct of *image schemas*, defined as recurrent, embodied patterns of experience such as CONTAINER, PATH, or BALANCE (Lakoff, 1987; Johnson, 1987). Image schemas represent particularly fundamental instances of schematic structure, combining a high degree of abstraction with direct grounding in sensorimotor experience. Yet, as Tuggy (2010) observes, their theoretical status remains complex: they are at once highly abstract and deeply embodied. This inherent tension epitomizes one of the central challenges for cognitive linguistics: accounting for how broad, deeply entrenched conceptual patterns interface with the more situated, context-dependent instantiations that emerge in language use and meaning construction.

Taken together, these frameworks reveal that specificity and generality (or schematicity) in cognitive linguistic research (and especially in theoretical cognitive linguistics) are not marginal notions but organizing principles of conceptual structure and linguistic meaning. They shape how categories are formed, how linguistic expressions are interpreted, and how abstraction is achieved through language. What remains underexplored, however, is how these well-established theoretical distinctions can be operationalized into metrics of specificity and concreteness and empirically (quantitatively!) tested within the context of embodied semantics.

## 4 Abstract vs. concrete and abstract vs. specific

The notion of *abstraction* has long been central to philosophy, psychology, and linguistics, yet its definition remains unsettled. The Latin root *abstrahere* (“to pull away from”) evokes a “pulling off” movement without specifying what is pulled out from what. In fact, there are two main ways in which we can define this mechanism. In the APA Dictionary of Psychology (VandenBos, 2007), abstraction refers to multiple sense, notably:

1. The formation of general ideas or concepts (e.g., *fish, hypocrisy*);

2. The representation of intangible concepts (e.g., *beauty, justice*).

Hence, ABSTRACT can be intended as the opposite of SPECIFIC (*animal* vs. *fish*, or *justice* vs. *restorative justice*) or as the opposite of CONCRETE (*beauty* vs. *necklace*).

A recent collective endeavor involving 54 authors who drafted, refined, provided feedback, and ultimately endorsed or disagreed with a set of definitions of semantic constructs, defined abstraction as “*the process of forming general ideas or concepts by extracting similarities and general tendencies from direct experience, language, or other concepts”* (Reilly et al., 2025: 250). Remarkably, this formulation received unanimous endorsement from all 54 co-authors involved in the initiative. Most importantly, in this glossary abstraction and abstractness are treated as distinct entries (as suggested by Dancygier and Sweetser, 2014 as well as by Borghi and Binkofski, 2014), precisely to avoid conflating the two notions of abstraction (that is, as the opposite of specificity, and as the opposite of concreteness).

The relationship between concreteness/abstractness and specificity/generality has remained an open question, as both dimensions are involved in the mechanisms of abstraction and are sometimes confused and conflated in the scientific literature, as noted by Bolognesi et al. (2020) under the intuition that a concept that seems very concrete is also very specific, while a concept that seems very abstract is also very general. In the context of the debate on the embodied nature of language, both these dimensions are fundamental. Yet, while concreteness (and its opposite abstractness) has been widely operationalized through experimental paradigms and metrics, as described in Section 2, specificity (and its opposite generality) has received far less attention and has been scarcely operationalized.

## 5 Disentangling the variables: concreteness and specificity

The research program carried out by the Abstraction research group in the past 3 years (and still ongoing) has investigated how specificity relates to and operates with concreteness, in language processing.

First, the Abstraction research group has released (small) datasets of specificity ratings in both Italian (Bolognesi and Caselli, 2023) and English (Ravelli et al., 2024), adopting the Best-Worst Scaling paradigm, a method in which annotators repeatedly select the “most” and “least” specific item from sets of 4 words, yielding reliable and fine-grained semantic judgments. These studies also showed the independent role that specificity has over word processing, above and beyond concreteness. In parallel, the group has explored different strategies to expand the resources available for studying specificity/generality. One approach involves approximating human specificity judgments with Large Language Models (LLMs) (Ravelli and Bolognesi, 2024), while another relies on production tasks, where participants are asked to generate word ladders spanning the generality–specificity relation (Villani et al., 2024; under review).

The first line of empirical evidence on the role of specificity on language processing comes from original word processing experiments conducted on a dataset of words that have been carefully selected to cover the spectrum of concreteness and specificity ranges. Using semantic decision tasks in Italian, Lamarra and colleagues demonstrated a robust specificity effect: words denoting more fine-grained categories are processed faster than their more general counterparts. In the task, participants were asked to classify concepts into either referring to a concrete referent or abstract. Independently from their concreteness, highly specific concepts were processed and categorized faster than highly general concepts, both concrete and abstract (Lamarra et al., 2025). A concreteness effect was also reported but, crucially, the two effects do not interact, supporting the claim that concreteness and specificity constitute distinct dimensions of semantic representation. Curiously, the effect of specificity on word processing (and likewise that of concreteness) were not found in lexical decision latencies in which participants were simply asked to indicate whether a string of letters presented on the screen was a genuine word or a non-word.

Moving beyond single-word tasks, the group has explored how concreteness and specificity shape conversational dynamics. Controlled dialogue studies revealed that sentences containing abstract terms tend to elicit curiosity and uncertainty, encouraging interlocutors to continue the exchange, while sentences framed with concrete terms cue certainty and conversational closure (Mazzuca et al., 2025). However, the level of specificity also plays a role in shaping conversational dynamics: highly specific wording fosters perceptions of assertiveness and closure, whereas more general terms invite curiosity and elaboration and expansion of the topic (Lamarra et al., in prep.). This last finding, however, has been observed on concrete (general and specific) concepts only. For abstract concepts there seems to be no difference in their processing time: both abstract general and abstract specific concepts require on average more time than concrete concepts.

On a different line of research, distributional investigations provided additional evidence for the independent role of specificity from concreteness in explaining contextual variability. Traditional accounts have suggested that the concreteness effect may be due to the higher availability of contextual information for concrete vs. abstract words (Schwanenflugel and Shoben, 1983; Schwanenflugel et al., 1988; Schwanenflugel and Stowe, 1989; Schwanenflugel, 1991). It has been argued that abstract words occur in broader and more diverse linguistic contexts than concrete ones. However, by introducing specificity into the framework, Rambelli and Bolognesi (2023, 2024) show that it is primarily specificity, rather than concreteness, that predicts distributional contextual breadth and density. Across both Italian and English corpora, more specific nouns tend to appear in narrower contexts, whereas more general nouns disperse across a wide range of contexts, regardless of their degree of concreteness. What earlier studies attributed to concreteness effects may therefore be more accurately understood as a function of semantic and taxonomic granularity or, as we labeled: specificity.

The Abstraction group has also explored a mechanism linked to the perceived specificity of a word as determined by its contextual use. In classic linguistic research, this phenomenon is known as *genericity* (Carlson and Pelletier, 1995), and it is defined as the extent to which a linguistic label applies to members of a category within a given context. For example, in the sentence *mosquitoes fly* the predication holds true for nearly all exemplars of the category, whereas in *mosquitoes bite* the predication applies only to female exemplars (roughly 50% of all mosquitoes), and *mosquitoes carry malaria* refers to a very limited subgroup (around 3% of all mosquitoes). Accordingly, the scope of the category *mosquitoes* expands or contracts in speakers' minds depending on the predicate (*fly, bite, carry malaria*). Traditional accounts have tended to treat this variability in binary terms, distinguishing between kind-referring and instance-referring uses (Krifka et al., 1995). By contrast, Collacciani et al. (2024a) proposed a paradigm and a dataset that operationalize the intuition of genericity of a word in context along two non-binary, but continuous dimensions: inclusiveness, reflecting degrees of generality/specificity, and abstractness, reflecting the concreteness/abstractness continuum. Their findings indicate that binary contrasts between generic and non-generic uses cannot capture the graded nature of meaning, and that both, specificity/generality and concreteness/abstractness contribute to the perception of how broad or narrow a category is intended, within a given context.

The group has also extended its investigation to comparisons between humans and LLMs, highlighting important differences in how humans and LLMs are sensitive to quantification, namely, to the linguistic and conceptual mechanisms by which expressions denote quantities over individuals, sets, or events, and establish scope relations within sentence structure (Lazaridou-Chatzigoga, 2019; Murphy, 2004; Pelletier, 2009). For instance, taking the *mosquitoes* example above, humans would choose the quantifier *few*, in front of the sentence *mosquitoes carry malaria*. They would not choose *most*, or *all*. Results show that while humans rely on pragmatic reasoning and world knowledge to flexibly calibrate generalizations, LLMs often under- or over-estimate such gradience (Collacciani et al., 2024b). Moreover, although LLMs can approximate human specificity ratings (Ravelli and Bolognesi, 2024; Puccetti et al., 2025), their performance is inconsistent across tasks and model types, and they show clear limitations in compositional generalization, such as extending relational patterns to novel compounds (e.g., from *olive oil* to *banana oil*; Rambelli et al., 2024). These findings align with broader evidence on differences in generalization between humans and machines (Ilievski et al., 2025), suggesting that while LLMs may be fairly reliable as scalable proxies for specificity judgments within the BWS methodological setting, they fall short of capturing the flexible, context-sensitive, and creative generalizations that characterize human abstraction in natural language use.

To conclude, this growing body of research shows that concreteness and specificity are best conceived as independent dimensions of conceptual representation, each contributing to the mechanisms of abstraction. Their distinct effects on processing, discourse, and contextual variability suggest that a fuller understanding of embodiment also requires examining how these dimensions interact. The next step of this paper, and the central concern of this special issue, is to set the ground for a new agenda in which it is investigated how concreteness and specificity jointly shape the embodiment of language.

## 6 Exploring the embodied nature of language referring to concrete, abstract, specific and general concepts: toward new pathways for research

What happens to the embodiment of concrete concepts once specificity is taken into account? For example, does the sensorimotor system become engaged to the same extent when processing a broad concept such as *food* as when processing more specific categories such as *dessert, ice cream*, or even *Double Caramel Magnum*? All of these terms, in the end, refer to tangible entities for which taste constitutes the primary sensory modality, yet their degree of specificity may shape both the manner and intensity with which they are grounded in perceptual experience. Superordinate categories indeed seem to be embodied in a weaker, less vivid, and more schematic fashion, typically invoking bodily schemas. This suggests a form of abstraction that emerges through the loss of perceptual distinctiveness, whereby embodiment is effectively compressed.

These considerations align with broader discussions of higher-order cognition within embodied frameworks described in the previous sections and summarized for instance by Lakoff and Johnson (1999), who posits that reasoning, categorization, and abstraction do not arise independently of bodily experience, but instead through the graded re-use of perceptual and motor resources. Nonetheless, empirical investigations remain limited, particularly those that explicitly treat concreteness and specificity as distinct but correlated theoretical dimensions.

A similar issue arises with abstract concepts: while some domains, such as *religion*, afford a relatively rich hierarchical taxonomy of hypernyms and hyponyms, others such as *beauty* or *time* seem to afford shorter taxonomies. Do these differences in taxonomic depth affect the degree to which and the mode in which the grounding is achieved? Are abstract concepts grounded differently as a function of their specificity? Do metaphorical mappings, situated simulations and emotional and social experiences contribute in the same way to explain the embodiment of abstract concepts expressed at different levels of abstraction? These, so far, remain open empirical questions. In addition to the contributions of the Abstraction research group, recent studies have begun to tackle these issues by creating resources that can be used to measure specificity and therefore prepare stimuli for empirical studies (e.g., Muraki and Pexman, under review) and by conducting experimental research (e.g., Greene and Rohan, 2025). Yet, significant progress is still needed toward a unified framework that embeds language-driven abstraction processes within embodied models. Such an approach would eventually help explain to what extent higher-order conceptual representations arise and become encoded in language, and to what extent they are driven and constructed by language.

To conclude and summarize, if concreteness has so far been treated as the primary predictor of sensorimotor grounding, specificity must now be recognized and operationalized into experimental studies alone and in interaction with concreteness. This reconceptualization expands the scope of embodied theories of language, which can no longer rely solely on the concrete-abstract dichotomy, but must also account for taxonomic granularity as a crucial determinant of how words are grounded in perception, action, and communicative experience. The recognition of specificity as a decisive factor in embodiment opens important avenues for research, and the time is ripe to consider the dimensions of concreteness and specificity as separated but related variables, to better understand how they jointly shape the mechanisms of abstraction and conceptual grounding.

In methodological terms, introducing specificity as an independent dimension encourages a re-analysis of existing datasets, where concreteness ratings alone may obscure crucial differences between concrete stimuli (maybe also quite specific) and abstract ones (maybe also quite general). It also suggests designing new experiments that manipulate both, concreteness and specificity, testing predictions about sensorimotor recruitment.

Looking ahead, advancing this line of inquiry requires a broad, interdisciplinary effort. Linguistics, philosophy, cognitive science, and neuroscience are essential for uncovering how language, perception, and neural mechanisms jointly support the embodiment of concepts at varying levels of abstraction. At the same time, collaborations with computer science and artificial intelligence hold the promise of developing computational models that can simulate and test hypotheses about embodied abstraction, offering valuable tools for both theoretical refinement and empirical validation. Only by bridging these domains can we move toward a comprehensive understanding of how the human mind integrates concreteness and specificity in conceptual grounding and, by extension, explore how artificial minds might replicate or diverge from these mechanisms. This convergence of disciplines thus represents not only a fertile pathway for future research, but also a call to collectively rethink embodiment and abstraction as core principles of cognition across natural and artificial systems.
